# AKR1B10 expression characteristics in hepatocellular carcinoma and its correlation with clinicopathological features and immune microenvironment

**DOI:** 10.1038/s41598-024-62323-5

**Published:** 2024-05-27

**Authors:** Li-Na Ma, Yan Ma, Xia Luo, Zi-min Ma, Li-Na Ma, Xiang-Chun Ding

**Affiliations:** 1 Ningxia Medical University, Yinchuan, 750004 Ningxia China; 2 Department of Infectious Disease, General Hospital of Ningxia Medical University, 804 Shengli South Street, Xingqing District, Yinchuan, 750004 Ningxia China; 3 Xinasheng Biotech of Ningxia, Yinchuan, 750004 Ningxia China

**Keywords:** Hepatocellular carcinoma, AKR1B10, Clinical-pathological features, Survival prognosis, Immune microenvironment, Regulatory mechanisms, Cancer microenvironment, Protein function predictions, Prognostic markers, Liver cancer

## Abstract

Hepatocellular carcinoma (HCC) represents a major global health threat with diverse and complex pathogenesis. Aldo–keto reductase family 1 member B10 (AKR1B10), a tumor-associated enzyme, exhibits abnormal expression in various cancers. However, a comprehensive understanding of AKR1B10's role in HCC is lacking. This study aims to explore the expression characteristics of AKR1B10 in HCC and its correlation with clinicopathological features, survival prognosis, and tumor immune microenvironment, further investigating its role and potential regulatory mechanisms in HCC. This study conducted comprehensive analyses using various bioinformatics tools and databases. Initially, differentially expressed genes related to HCC were identified from the GEO database, and the expression of AKR1B10 in HCC and other cancers was compared using TIMER and GEPIA databases, with validation of its specificity in HCC tissue samples using the HPA database. Furthermore, the relationship of AKR1B10 expression with clinicopathological features (age, gender, tumor size, staging, etc.) of HCC patients was analyzed using the TCGA database's LIHC dataset. The impact of AKR1B10 expression levels on patient prognosis was evaluated using Kaplan–Meier survival analysis and the Cox proportional hazards model. Additionally, the correlation of AKR1B10 expression with tumor biology-related signaling pathways and tumor immune microenvironment was studied using databases like GSEA, Targetscan, and others, identifying microRNAs (miRNAs) and long non-coding RNAs (lncRNAs) that regulate AKR1B10 expression to explore potential regulatory mechanisms. Elevated AKR1B10 expression was significantly associated with gender, primary tumor size, and fibrosis stage in HCC tissues. High AKR1B10 expression indicated poor prognosis and served as an independent predictor for patient outcomes. Detailed mechanism analysis revealed a positive correlation between high AKR1B10 expression, immune cell infiltration, and pro-inflammatory cytokines, suggesting a potential DANCR-miR-216a-5p-AKR1B10 axis regulating the tumor microenvironment and impacting HCC development and prognosis. The heightened expression of AKR1B10 in HCC is not only related to significant clinical-pathological traits but may also influence HCC progression and prognosis by activating key signaling pathways and altering the tumor immune microenvironment. These findings provide new insights into the role of AKR1B10 in HCC pathogenesis and highlight its potential as a biomarker and therapeutic target.

## Introduction

Hepatocellular carcinoma (HCC) is one of the most common malignancies globally, accounting for 75–85% of primary liver cancers. As a significant public health issue, especially in East Asia, such as China, HCC presents a median survival of only 6–10 months^1^. Liver cirrhosis, a key pathological foundation for HCC, is a common cause of death in patients with cirrhosis^2^. Factors promoting cirrhosis, including viral infections, non-alcoholic fatty liver disease (NAFLD), chronic alcoholism, aflatoxin exposure, and genetic factors, are also recognized risk factors for HCC progression^3^. With increasing global incidence of NAFLD, HCC cases due to this etiology are expected to rise^4^. Despite advancements in interventional and targeted therapies improving HCC diagnosis and treatment, the high heterogeneity of HCC, difficulties in early diagnosis, and development of acquired drug resistance, coupled with limited understanding of molecular mechanisms of hepatocellular malignancy, result in a generally low five-year survival rate below 15% for advanced HCC patients^5^. Therefore, exploring the pathogenesis of liver cancer, understanding the mechanisms of hepatocellular malignancy, and identifying new potential biomarkers remain key directions in addressing this global challenge.

The aldo–keto reductase (AKR) protein superfamily, a group of NADPH-dependent oxidoreductases, plays a crucial role in the metabolism of carbohydrates, steroids, glycyrrhizin, other endogenous aldehydes and ketones, and exogenous compounds. Dysregulated expression and functional disorder of these enzymes can lead to various diseases, including disrupted cell metabolic pathways, impaired detoxification functions of carbon-based compounds, and activation of carcinogens^6^. AKR1B10, a vital member of the AKR family, not only functions in reducing aliphatic and aromatic aldehydes but also plays a significant role in the biological processes of various cancers, including lung (including squamous cell carcinoma and smoking-related adenocarcinoma)^7^, oral squamous cell carcinoma^8^, breast cancer^9^, and pancreatic cancer^10^. Moreover, AKR1B10 has been recognized as a key regulatory factor in the occurrence and development of HCC. Studies at the cellular and tissue levels have confirmed its high expression in HCC, and it has been suggested as an important biomarker for the differential diagnosis of early HCC^11,12^. However, its expression in HCC clinical samples varies according to the stage of liver cancer, being significantly overexpressed in relatively early tumor stages with potential cirrhosis or viral hepatitis and downregulated in late stages with lower differentiation grades^13,14^. This indicates that the expression of AKR1B10 in hepatocellular carcinoma is unstable. Furthermore, it has been found to regulate the characteristics of liver cancer stem cells, including self-renewal, tumor-forming ability, and expression of liver cancer stem cell markers, by modulating the activity of the AP-1 complex within hepatocellular carcinoma cells and apoptotic cascade signaling pathways, leading to reduced sensitivity to sorafenib and the development of drug resistance^15^.

Despite AKR1B10 being considered a key factor in inducing cancer and acquired drug resistance, its expression characteristics in HCC and its correlation with clinicopathological features and tumor immune microenvironment have not been detailed, and its impact on clinical prognosis in HCC remains controversial^16^. Hence, this study aims to investigate the expression level of AKR1B10 in HCC through bioinformatics analysis techniques and analyze its correlation with clinical characteristics of HCC patients, such as age, gender, tumor size, grading, and survival status, to validate whether the clinical prognostic status indeed correlates with differential expression of AKR1B10. Additionally, this study will explore the correlation between AKR1B10 expression and immune cell infiltration in HCC, uncover potential signaling pathways involved, and identify LncRNA–miRNA networks regulating AKR1B10 expression, aiming to reveal its unique expression patterns and possible biological functions in HCC, providing new targeted strategies for the diagnosis and treatment of hepatocellular carcinoma.

## Materials and methods

### Data acquisition and differential gene expression analysis

This study utilized several bioinformatics tools and public databases, including the GEO database (https://www.ncbi.nlm.nih.gov/geo/), for identifying differentially expressed genes (DEGs) related to hepatocellular carcinoma (HCC). Screening out DEGS with |log2FC|> 1 and *P*-value < 0.05 as the standard. Through UpSet diagrams to visualize the cross differentially expressed genes were screened out from these datasets.

### AKR1B10 expression analysis at mRNA and tissue levels

The expression of AKR1B10 in tumor and adjacent normal tissues was analyzed using the TIMER (https://timer.cistrome.org/)^17^ and GEPIA (https://gepia2.cancer-pku.cn)^18^ databases. The Human Protein Atlas (HPA) (https://www.proteinatlas.org/)^19^ database was used to validate the specific expression of AKR1B10 in HCC tissue samples.

### Correlation analysis of AKR1B10 expression with clinicopathological features of HCC

Clinical and transcriptomic data from the TCGA-LIHC dataset were obtained from the UCSC database (https://xenabrowser.net/datapages/)^47^. HCC patients were categorized into high and low AKR1B10 expression groups based on the median expression level of AKR1B10 mRNA, to analyze its correlation with clinicopathological characteristics.

### Prognostic impact of AKR1B10 expression in HCC patients

Kaplan–Meier survival analysis and Cox proportional hazards model, using the GSCA Lite database (https://bioinfo.life.hust.edu.cn/web/GSCA)^20^, were employed to assess the association between AKR1B10 expression and patient prognosis.

### Gene enrichment analysis

Gene Set Enrichment Analysis (GSEA) (https://software.broadinstitute.org/gsea/msigdb/) was conducted on the top 100 genes most strongly associated with AKR1B10 expression, extracted from the GEPIA databases (https://gepia2.cancer-pku.cn)^18^. For each analysis, gene set permutation was performed 1000 times. Gene sets with a normal *P*-value less than 5% and false discovery rate less than 25% were considered as significantly enriched.The Link Interpreter module in the LinkedOmics database(https://www.linkedomics.org)^21^ was used to explore co-expressed genes related to LIHC survival were obtained from the TCGA.These genes were enriched by Gene Ontology (GO) (including biological processes, cellular components and molecular function) and Kyoto Encyclopedia of Genes and Genomes (KEGG)^52^ pathway analyses using the DAVID database (https://david.ncifcrf.gov/home.jsp)^22^. A corrected P < 0.05 was determined to be statistically significant.

### Correlation analysis of AKR1B10 with immune cell infiltration and immune checkpoint-related genes

The study analyzed the correlation between AKR1B10 expression and tumor immune cell infiltration (TIIC), using the TIMER database (https://timer.cistrome.org/)^17^. It also explored the relationship between AKR1B10 and immune checkpoint-related genes in HCC.

### LncRNA–miRNA–AKR1B10 regulatory network prediction

Databases like miRDB (https://mirdb.org/)^23^, miRWalk (https://mirwalk.umm.uni-heidelberg.de/)^24^, and Targetscan (https://www.targetscan.org/)^25^ were used to predict miRNAs that bind upstream of AKR1B10, and LncBASE (https://diana.e-ce.uth.gr/lncbasev3)^26^, miRNet (https://www.mirnet.ca/miRNet/home.xhtml)^27^, and StarBASE (https://rnasysu.com/encori/)^28^ databases were employed to reverse-predict LncRNAs targeting these miRNAs.

### Statistical analysis

Data processing and analysis were conducted using GraphPad Prism 9.0 software. Continuous data were analyzed using the unpaired t-test or Wilcoxon rank-sum test, while categorical variables were analyzed using the chi-square test or Fisher's exact test. And *P* < 0.05 was considered statistically significant.

## Results

### Identification of significant differentially expressed genes (DEGs) related to hepatocellular carcinoma based on GEO database

**Figure 1:**
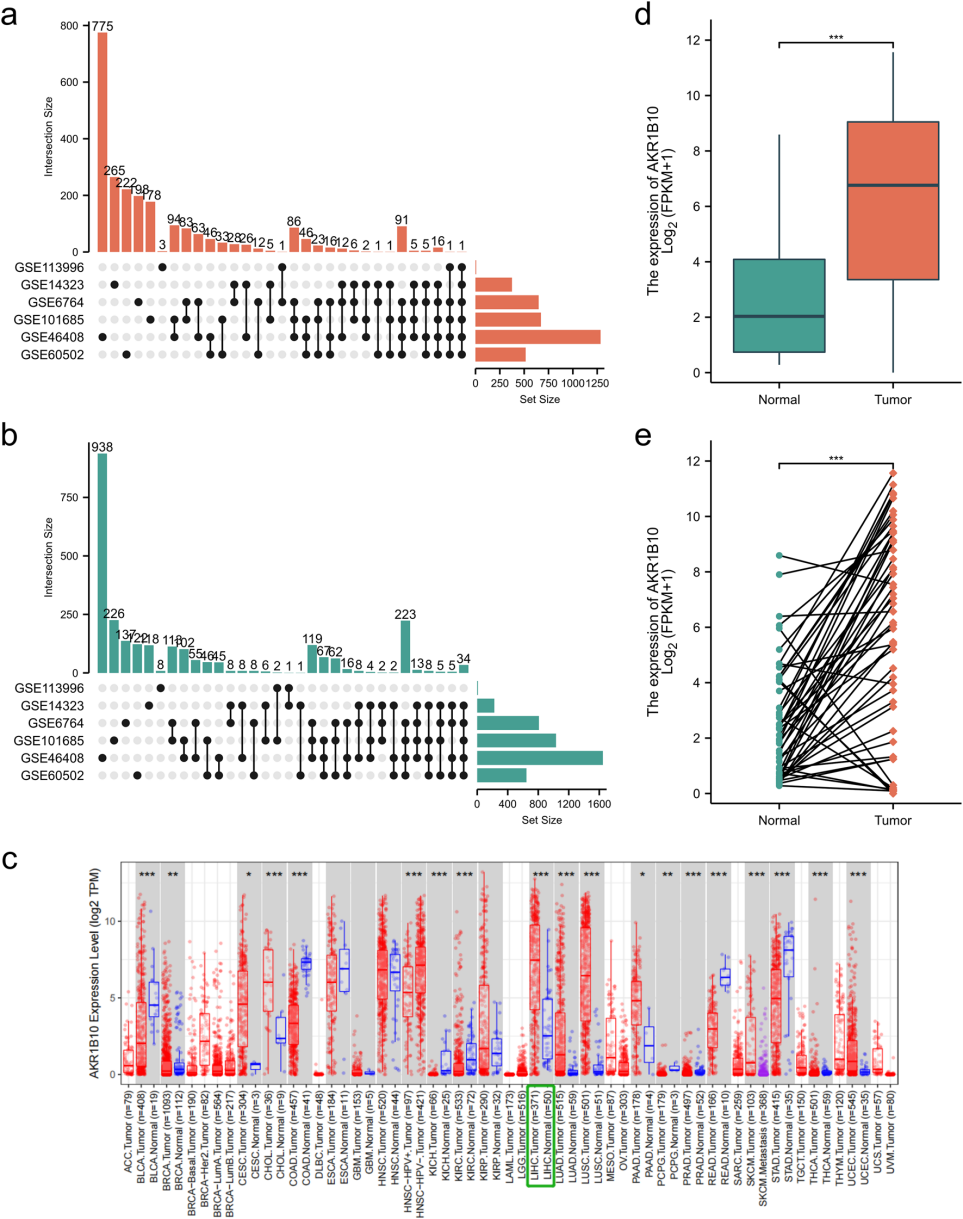
High expression of AKR1B10 in hepatocellular carcinoma: (**a**) significantly overexpressed differentially expressed genes in six datasets, (**b**) significantly underexpressed differentially expressed genes in six datasets, (**c**) expression of AKR1B10 in various cancers, (**d**) differential expression of AKR1B10 in liver cancer tissues compared to non-cancerous tissues in non-paired samples, (**e**) Differential expression of AKR1B10 in liver cancer tissues compared to non-cancerous tissues in paired samples.

Gene expression profiles from six liver cancer-related datasets (GSE14323, GSE46408, GSE60502, GSE6764, GSE101685, GSE113996) were retrieved from the GEO database (as shown in Table 1 for details). Using the online tool GEO2R (https://www.ncbi.nlm.nih.gov/geo/geo2r), DEGs between liver cancer tissues and non-cancerous tissues were screened, with *P*-value < 0.05 and |logFC|> 1.0 as the selection criteria. From GSE14323, a total of 777 DEGs were obtained, including 513 upregulated and 264 downregulated genes; from GSE46408, 2932 DEGs were found, with 1285 genes upregulated and 1647 downregulated; GSE60502 revealed 514 upregulated and 644 downregulated genes; GSE6764 yielded 1456 DEGs, comprising 648 upregulated and 808 downregulated genes; from GSE101685, a total of 1703 DEGs were obtained, including 671 upregulated and 1032 downregulated genes; GSE113996 dataset had 17 DEGs, with 6 upregulated and 11 downregulated. UpSet diagrams were constructed to visualize the intersection of these DEGs (as shown in Fig. 1a, b), identifying AKR1B10 as significantly upregulated across all six datasets, hence it was selected as the target gene for this study.

**Table 1:** Detailed information about GEO datasets.

GEO datasets	Platforms	Non-tumors	HCC tumors	AKR1B10-logFC
GSE14323^48^	GPL571	19	55	3.7119827
GSE46408^49^	GPL4133	6	6	4.1270174
GSE60502^50^	GPL96	18	18	2.2351984
GSE6764^51^	GPL570	10	35	6.0276762
GSE101685	GPL570	8	24	4.1254531
GSE113996	GPL16043	20	20	1.82717268

### High expression of AKR1B10 in hepatocellular carcinoma

The TIMER database was utilized to observe AKR1B10 expression levels across various cancer types. Pan-cancer analysis revealed that compared to non-cancerous tissues, AKR1B10 expression was significantly elevated in cholangiocarcinoma (CHOL), hepatocellular carcinoma (LIHC), lung adenocarcinoma (LUAD), lung squamous cell carcinoma (LUSC), prostate cancer (PRAD), and uterine endometrial cancer (UCEC). Moreover, in bladder urothelial carcinoma (BLCA), colon adenocarcinoma (COAD), kidney chromophobe (KICH), kidney renal clear cell carcinoma (KIRC), rectum adenocarcinoma (READ), and stomach adenocarcinoma (STAD), AKR1B10 expression levels showed a decreasing trend compared to normal tissues (Fig. 1c). Analysis using the GEPIA database, which combines TCGA and GTEx data, indicated that at the mRNA level, AKR1B10 expression in HCC was higher than in non-cancerous tissues, both in non-paired samples (Fig. 1d) and paired samples (Fig. 1e), consistent with the pan-cancer analysis results.

### Tissue-specific expression of AKR1B10 in hepatocellular carcinoma

**Figure 2:**
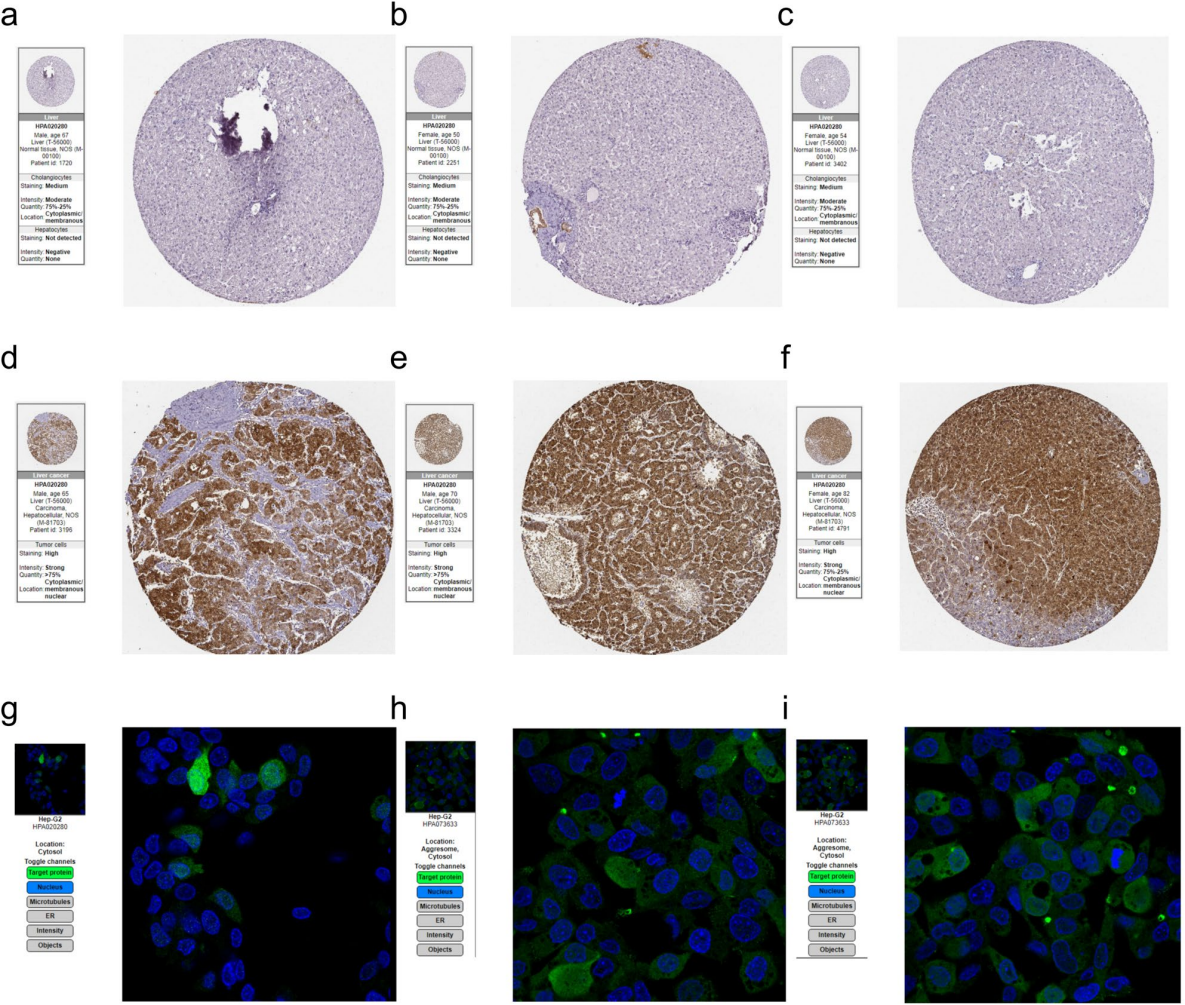
Expression of AKR1B10 at the Tissue Level in the HPA Database: (**a–c**) IHC shows no detection of AKR1B10 expression in normal liver tissues, (**d–f**) High levels of AKR1B10 expression observed in liver cancer tissues, (**g–i**) IF demonstrates the localization of AKR1B10 in HepG2 liver cancer cells.

To further confirm the expression at the tissue level, quantitative analysis of tissue immunohistochemistry (IHC) from the Human Protein Atlas (HPA) database indicated high expression of AKR1B10 in hepatocellular carcinoma tissues and almost no expression in healthy liver tissues (Fig. 2a–f). Additionally, subcellular localization under immunofluorescence (IF) staining showed that AKR1B10 is primarily located in the cytoplasm of HepG2 cells (Fig. 2g–i), consistent with its established function as an enzyme catalyzing oxidation–reduction reactions.

### Correlation between AKR1B10 expression level and clinicopathological features

**Figure 3:**
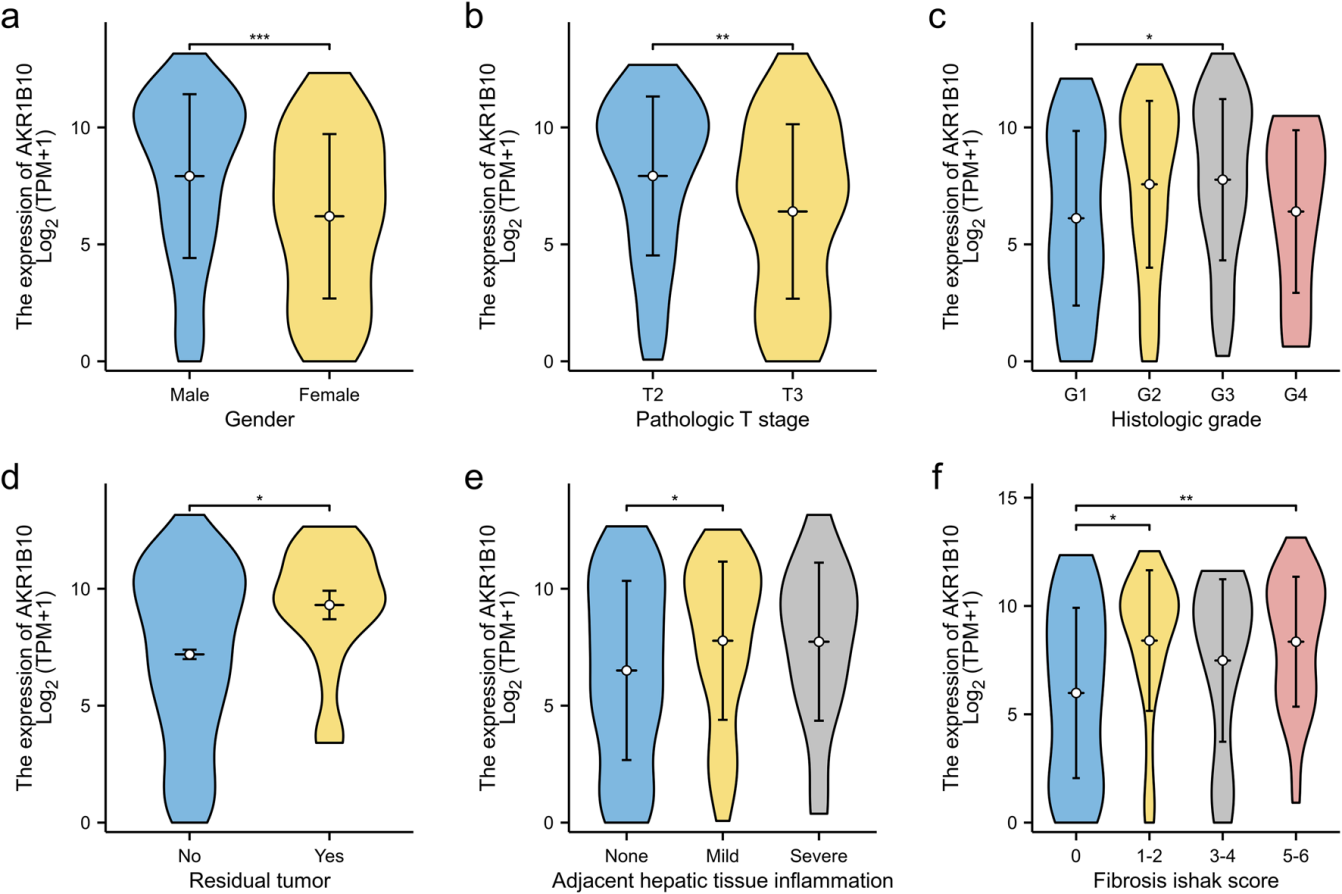
Correlation between AKR1B10 expression and clinicopathological features in hepatocellular carcinoma patients: (**a**) correlation with gender, (**b**) correlation with T stage, (**c**) correlation with tissue grading, (**d**) correlation with postoperative tissue margin status, (**e**) correlation with inflammation in adjacent tissues, (**f**) correlation with Ishak fibrosis score.

Based on the median expression level of AKR1B10 mRNA, hepatocellular carcinoma patients from the TCGA-LIHC cohort were divided into high and low expression groups for correlation analysis with clinicopathological features (Table 2). Statistical data indicated that the expression level of AKR1B10 was significantly correlated with gender, pathological staging, tissue grading, status of surgical margins, inflammation in adjacent hepatic tissues, and the degree of fibrosis. Specifically, higher expression was observed in tumor tissues of male patients at an early stage of disease, with a higher degree of tissue differentiation, more severe fibrosis, inflammation in adjacent liver tissues, or residual lesions at postoperative margins (Fig. 3a–f).

**Table 2:** Correlation between AKR1B10 expression and clinicopathological features of patients with hepatocellular carcinoma.

Characteristics	Low expression of AKR1B10	High expression of AKR1B10	*P*	Characteristics	Low expression of AKR1B10	High expression of AKR1B10	*P*
N	187	187		n	187	187	
Age, n (%)			0.194	Histologic grade, n (%)			0.860
≤ 60	95 (25.5%)	82 (22%)		G1 + G2	116 (31.4%)	117 (31.7%)	
> 60	92 (24.7%)	104 (27.9%)		G3 + G4	69 (18.7%)	67 (18.2%)	
Gender, n (%)			** < 0.001**	Vascular invasion, n (%)			1.000
Female	79 (21.1%)	42 (11.2%)		No	104 (32.7%)	104 (32.7%)	
Male	108 (28.9%)	145 (38.8%)		Yes	55 (17.3%)	55 (17.3%)	
Pathologic stage, n (%)			**0.044**	Adjacent hepatic tissue inflammation, n (%)			**0.011**
Stage I + stage II	124 (35.4%)	136 (38.9%)		No	72 (30.4%)	46 (19.4%)	
Stage III + stage IV	54 (15.4%)	36 (10.3%)		Yes	53 (22.4%)	66 (27.8%)	
T stage, n (%)			**0.039**	Child–Pugh grade, n (%)			0.572
T1 + T2	130 (35.0%)	148 (39.9%)		A	106 (44.0%)	113 (46.9%)	
T3 + T4	55 (14.8%)	38 (10.2%)		B	9 (3.7%)	12 (5.0%)	
N stage, n (%)			0.681	C	1 (0.4%)	0 (0.0%)	
N0	132 (51.2%)	122 (47.3%)		Fibrosis Ishak score, n (%)			0.009
N1	3 (1.2%)	1 (0.4%)		0	47 (21.9%)	28 (13.0%)	
M stage, n (%)			0.573	1 + 2	10 (4.7%)	21 (9.8%)	
M0	139 (51.1%)	129 (47.4%)		3 + 4	12 (5.6%)	16 (7.4%)	
M1	1 (0.4%)	3 (1.1%)		5 + 6	33 (15.3%)	48 (22.3%)	

Significant values are in bold.

### Impact of AKR1B10 expression on the prognosis and survival of patients with hepatocellular carcinoma

**Figure 4:**
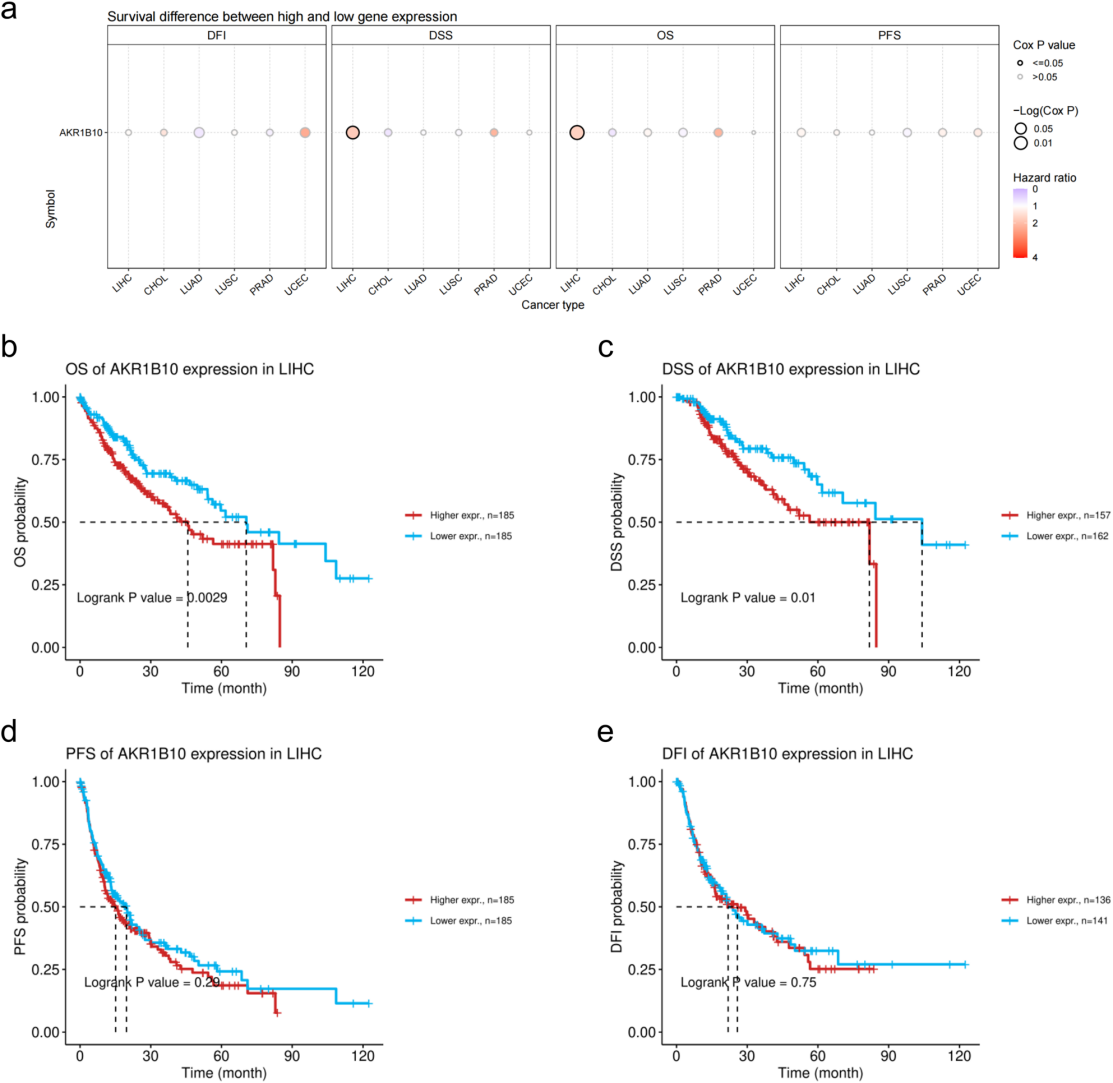
Correlation between AKR1B10 expression and prognosis in liver cancer patients in the GSCA database: (**a**) correlation of AKR1B10 with the prognosis of patients with six types of cancer: CHOL, LIHC, LUAD, LUSC, PRAD, UCEC; (**b–e**) correlation between the prognosis of liver cancer patients and the expression level of AKR1B10.

Previous studies have established high expression of AKR1B10 in several cancers, including cholangiocarcinoma, hepatocellular carcinoma, lung adenocarcinoma, lung squamous cell carcinoma, prostate cancer, and uterine endometrial cancer, compared to adjacent normal tissues. An analysis using the GSCA database examined the relationship between AKR1B10 expression and the prognosis of patients with these six types of tumors from the TCGA + GTEx dataset. The results indicated that although AKR1B10 is highly expressed in various tumors, it significantly affects the prognosis of hepatocellular carcinoma patients only (Fig. 4a). Specifically, high levels of AKR1B10 significantly impact the overall survival (OS) and disease-specific survival (DSS) of HCC patients, while progression-free survival (PFS) and disease-free interval (DFI) are not affected by AKR1B10 expression (Fig. 4b–e).

Moreover, Cox regression analysis was applied to identify factors affecting the prognosis of liver cancer patients. As shown in Table 3, univariate analysis revealed that tumor status (HR = 2.317, *P* < 0.001), pathological grading (HR = 2.504, *P* < 0.001), T stage (HR = 2.598, *P* < 0.001), M stage (HR = 4.077, *P* = 0.017), and AKR1B10 expression (HR = 1.711, *P* = 0.002) were significantly related to overall survival (OS) of liver cancer patients. Further multivariate analysis suggested that AKR1B10 expression (HR = 1.908, *P* = 0.006) indeed serves as an independent prognostic factor.

**Table. 3:** Univariate and multivariate Cox regression analysis of overall survival in LIHC patients from TCGA.

Characteristics	Total (N)	Univariate analysis		Multivariate analysis	
		Hazard ratio (95% CI)	*P* value	Hazard ratio (95% CI)	*P* value
Age	373				
≤ 60	177	Reference			
> 60	196	1.205 (0.850–1.708)	0.295		
Gender	373				
Female	121	Reference			
Male	252	0.793 (0.557—1.130)	0.200		
Tumor status	354				
Tumor free	202	Reference		Reference	
With tumor	152	2.317 (1.590–3.376)	** < 0.001**	1.918 (1.201–3.063)	**0.006**
Pathologic stage	349				
Stage I + stage II	259	Reference		Reference	
Stage III + Stage IV	90	2.504 (1.727–3.631)	** < 0.001**	1.608 (0.219–11.779)	0.640
T stage	370				
T1 + T2	277	Reference		Reference	
T3 + T4	93	2.598 (1.826–3.697)	** < 0.001**	1.724 (0.233–12.725)	0.594
M stage	272				
M0	268	Reference		Reference	
M1	4	4.077 (1.281–12.973)	**0.017**	0.901 (0.211–3.854)	0.889
Child–Pugh grade	240				
A	218	Reference			
B + C	22	1.643 (0.811–3.330)	0.168		
Vascular invasion	317				
No	208	Reference			
Yes	109	1.344 (0.887–2.035)	0.163		
AKR1B10	373				
Low	187	Reference		Reference	
High	186	1.711 (1.198–2.444)	**0.003**	1.908 (1.199–3.034)	**0.006**

Significant values are in bold.

### AKR1B10 may influence the biological progression of HCC through various signaling pathways

**Figure 5:**
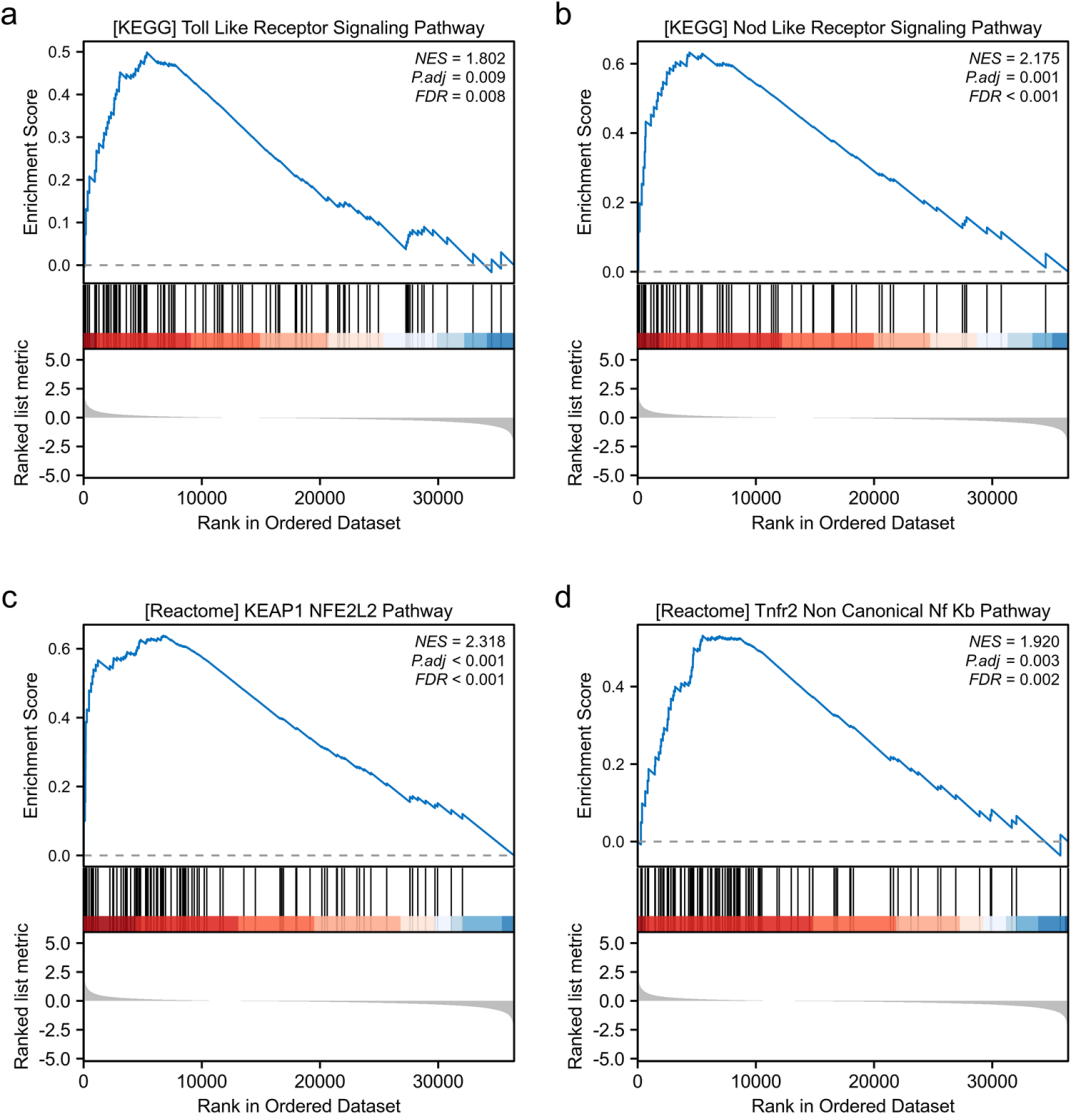
GSEA functional enrichment analysis of genes associated with AKR1B10 (**a–d**).

From the TCGA dataset, we extracted the top 100 genes most strongly associated with AKR1B10 and conducted Gene Set Enrichment Analysis (GSEA). This identified four pathways with significant statistical relevance:

KEGG_TOLL_LIKE_RECEPTOR_SIGNALING_PATHWAY, KEGG_NOD_LIKE_RECEPTOR_SIGNALING_PATHWAY, REACTOME_KEAP1_NFE2L2_PATHWAY, and REACTOME_TNFR2_NON_CANONICAL_NF_κB_PATHWAY (Fig. 5a–d). Therefore, AKR1B10 may influence the biological processes of hepatocellular carcinoma through regulating the release of inflammatory factors and cellular metabolism, although the specific mechanisms still require validation through basic experimental research.

### GO/KEGG functional enrichment analysis of AKR1B10-related genes in LIHC

**Figure 6:**
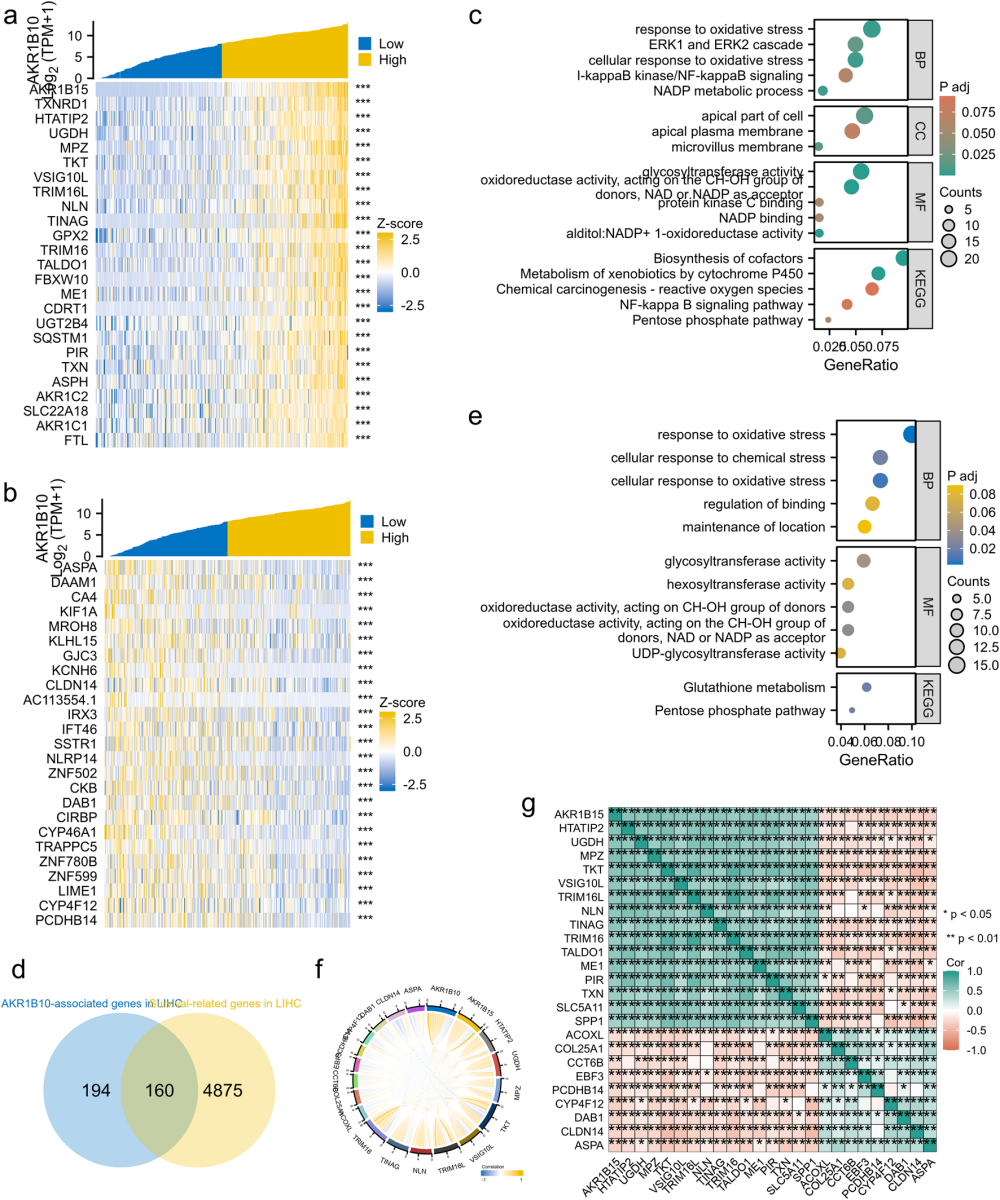
AKR1B10 functional clustering and interaction network analysis of AKR1B10-related genes: (**a**) heatmap showing the top 25 genes in LIHC that were positively related to AKR1B10, (**b**) heatmap showing the top 25 genes in LIHC that were negatively related to AKR1B10, (**c**) Gene Ontology (GO) term and Kyoto Encyclopedia of Genes and Genomes (KEGG) pathway analyses of AKR1B10-related genes in LIHC, (**d**) Venn diagram of AKR1B10-related genes and survival-related genes in LIHC, (**e**) GO term and KEGG pathway analyses of AKR1B10-related genes and survival-related genes in LIHC, (**f**) AKR1B10-survival-related gene interaction in chord diagram, (**g**) Gene coexpression matrix.

To further understand the biological function of AKR1B10 in hepatocellular carcinoma, we used the LinkRank module on the LinkedOmics website to detect the co-expression pattern of AKR1B10 in LIHC within the TCGA database. We selected the top 25 positively correlated genes (Fig. 6a) and the bottom 25 negatively correlated genes (Fig. 6b) for GO/KEGG enrichment analysis. The results revealed high enrichment in oxidation-related stress responses involving NADP+, biosynthesis of small molecules, and the NF-κB signaling pathway (Fig. 6c). To identify genes associated with AKR1B10 and significantly related to the prognosis of liver cancer, we intersected the top 354 genes most strongly correlated with AKR1B10 (absolute correlation coefficient ≥ 0.3) with 5035 upregulated survival-related genes in LIHC (p Cox < 0.05), resulting in 160 AKR1B10 prognostic-related genes (Fig. 6d). These 160 protein-coding genes may be potential genetic biomarkers for hepatocellular carcinoma patients. GO functional enrichment and KEGG pathway analysis of these 160 genes showed significant enrichment in processes such as regulation of redox reactions, glucose-6-phosphate metabolism, and astrocyte differentiation (Fig. 6e), all closely related to the development of hepatocellular carcinoma. Subsequent co-expression analysis of these prognostic-related genes identified their interactions, revealing a stronger enrichment network (Fig. 6f) and positive correlations (Fig. 6g), suggesting their potential as multi-gene biomarkers to predict survival prognosis in hepatocellular carcinoma patients.

### Correlation analysis between AKR1B10, immune cell infiltration, and immune checkpoint-related genes in liver cancer

**Figure 7:**
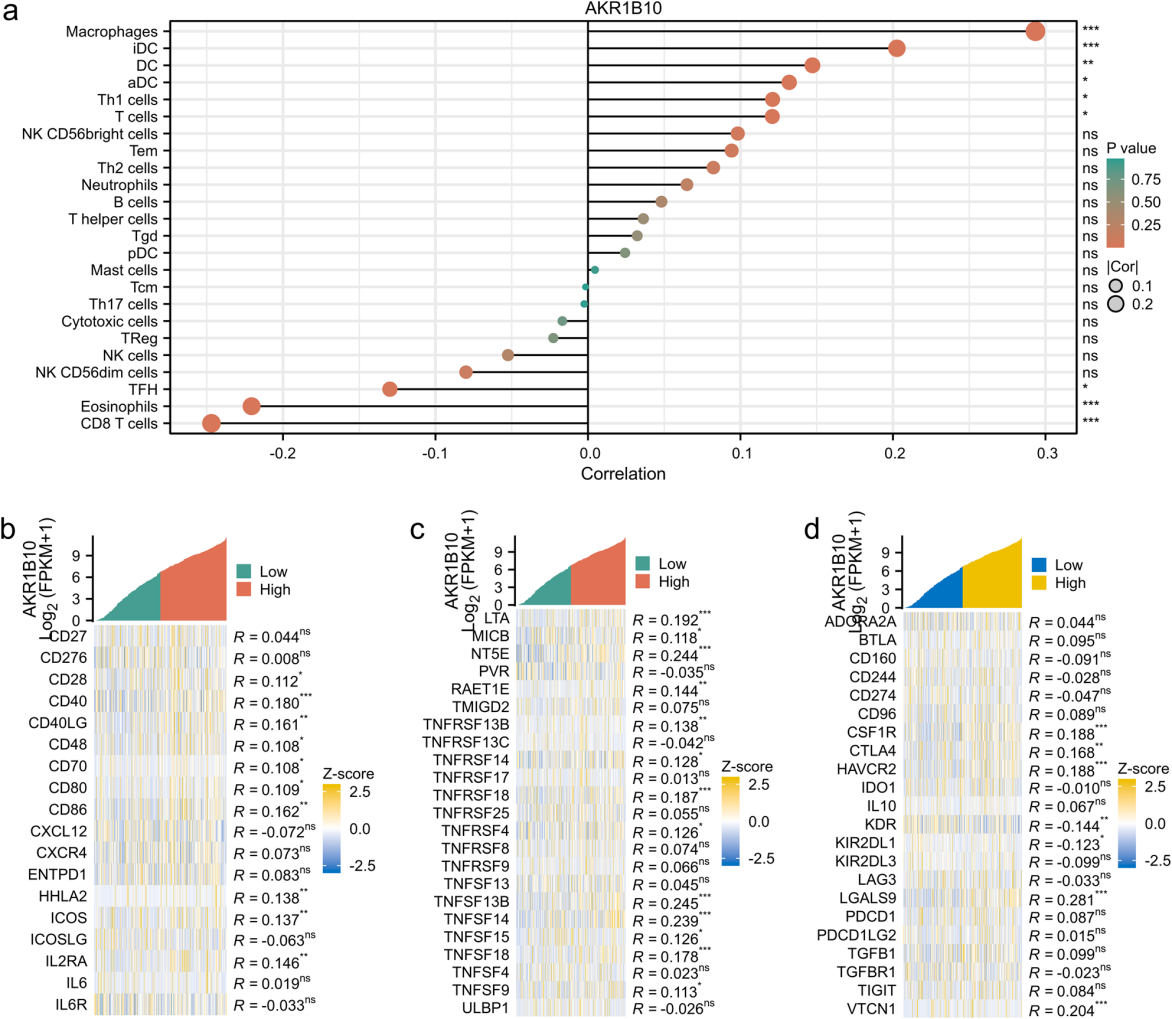
Analysis of the correlation between AKR1B10 and the tumor immune microenvironment: (**a**) analysis of tumor-related immune cell infiltration between high and low AKR1B10 expression groups based on public data, (**b,c**) analysis of the correlation between AKR1B10 and immune-promoting factors, (**d**) analysis of the correlation between AKR1B10 and immune-inhibitory factors.

Enrichment analysis suggested that AKR1B10 might modulate inflammatory factors, which are crucial components of the tumor microenvironment and affect tumor growth and development. To explore the correlation between AKR1B10 expression and tumor immune response, we used the TIMER database for further analysis. Significant correlations were found between AKR1B10 and tumor-infiltrating immune cells. Macrophages, activated dendritic cells, immature/mature dendritic cells, T cells, and Th1 cells showed positive correlation with AKR1B10 levels, while follicular helper T cells, eosinophils, and CD8+ T cells were negatively correlated (Fig. 7a). Additionally, we explored the relationship between AKR1B10 and immune regulatory factors by analyzing the expression levels of AKR1B10 and immune checkpoint-related genes (Fig. 7b–d). AKR1B10 showed significant correlations with most members of the Tumor Necrosis Factor Superfamily (TNFSF) and positively correlated with the immune checkpoint molecule CTLA-4. TNFSF can act as inflammatory mediators, activate NF-κB and MAPK signaling pathways through receptor binding, and promote inflammation. These findings reinforce the significant role of AKR1B10 in modulating the immune microenvironment and its impact on the development of liver cancer.

### AKR1B10's potential involvement in competitive endogenous RNA regulatory mechanisms

**Figure 8:**
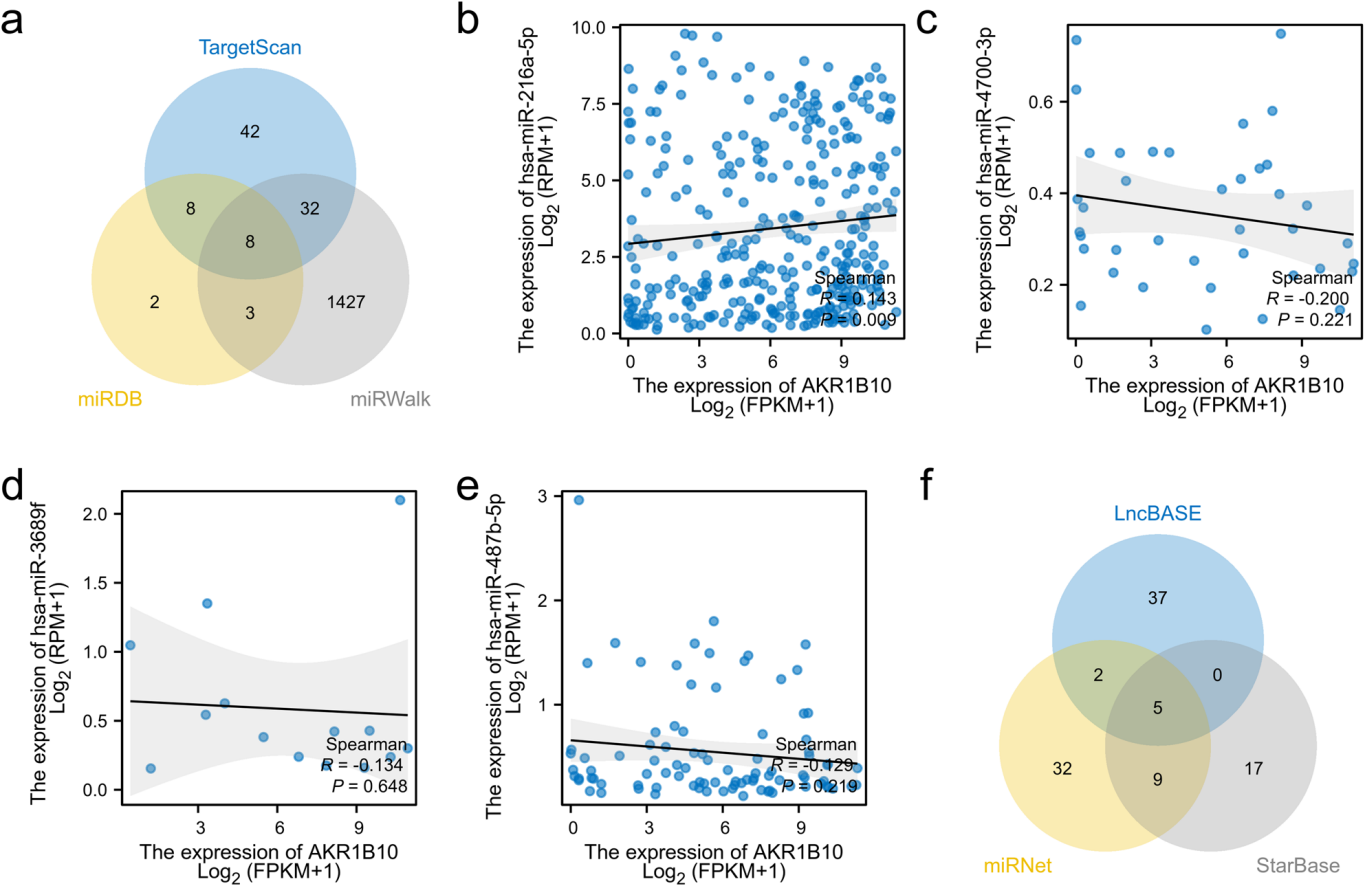
Predicted ceRNAs Interacting with AKR1B10: (**a**) miRNAs predicted from three datasets, (**b–e**) correlation analysis between AKR1B10 and miRNAs, (**f**) LncRNAs predicted from three datasets.

**Figure 9:**
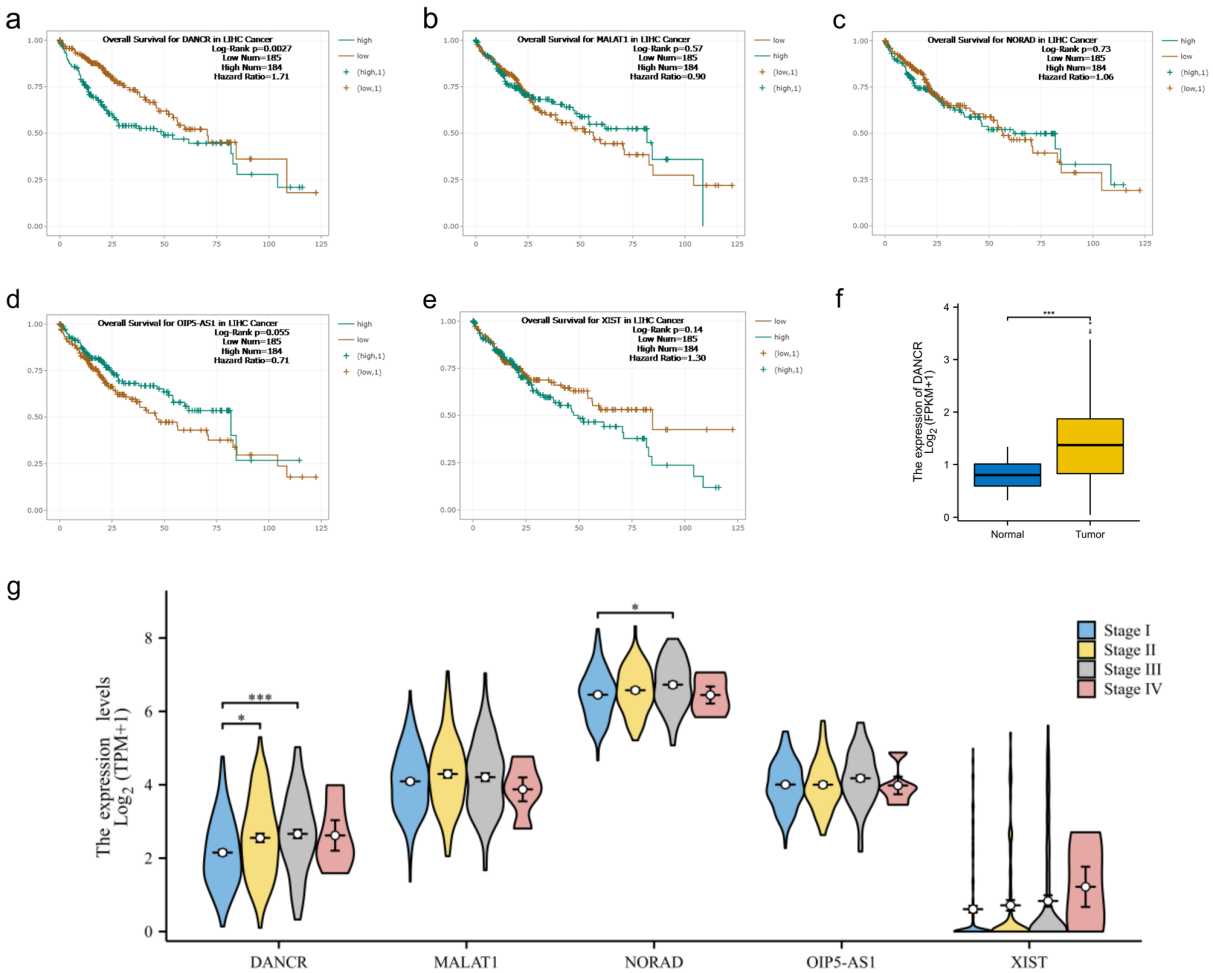
Correlation Analysis of LncRNAs in LIHC: (**a–e**) correlation analysis of the five predicted LncRNAs with the overall survival rate of liver cancer patients, (**f**) expression differences of DANCR in liver cancer tissues compared to non-cancerous tissues in unpaired samples.

To better understand the mechanism of action of AKR1B10 in the development and progression of hepatocellular carcinoma, we used three databases, miRDB, miRWalk, and Targetscan, to predict upstream miRNAs of AKR1B10. By intersecting the predicted genes, we identified 8 miRNAs regulating AKR1B10 (Fig. 8a), namely hsa-miR-216a-5p, hsa-miR-4298, hsa-miR-4700-3p, hsa-miR-3151-3p, hsa-miR-3689f, hsa-miR-5196-3p, hsa-miR-487b-5p, and hsa-miR-487a-5p. After excluding data with no expression in TCGA-LIHC and conducting a correlation analysis, only miR-216a-5p was found to be significantly correlated with AKR1B10 expression (p = 0.009) (Fig. 8b–e). Combining the prediction of miRNA targets, we used LncBASE, miRNet, and StarBASE databases to reverse predict upstream LncRNAs of miR-216a-5p, namely DANCR, MALAT1, NORAD, OIP5-AS1, and XIST (Fig. 8f). Analysis from Starbase on the influence of these five genes on the prognosis and survival of liver cancer patients showed that only DANCR was significantly related to the overall survival rate of liver cancer patients (Fig. 9a–e), and it was highly expressed in LIHC (Fig. 9f). In addition, we further studied the correlation between lncRNAs and different pathological stages of LIHC. The results showed that the expression level of DNACR was significantly correlated with higher pathological stage (p < 0.001), and indicated that DANCR may play a vital role in hepatocellular carcinoma progression. Given the above, we infer that AKR1B10 may exert its effect in hepatocellular carcinoma through the DANCR-miR-216a-5p-AKR1B10 regulatory network.

## Discussion

Hepatocellular carcinoma (HCC) is a highly malignant tumor that significantly contributes to the global cancer incidence rate. According to data from the International Agency for Research on Cancer of the World Health Organization, HCC is the fourth and fifth leading cause of cancer-related deaths in the age groups of 50–64 and 65–79 years, respectively^29^. HCC usually presents no obvious symptoms in its early stages, and the poor compliance of high-risk patients with monitoring leads to most patients being diagnosed in the late stage of the disease. Although the 5-year overall survival rate for patients diagnosed early and treated can reach up to 70%, the overall survival rate for liver cancer patients is only 19% after normalized analysis^30^. Alpha-fetoprotein (AFP), although recognized as a reliable marker for liver cancer screening and early diagnosis, numerous studies have shown that nearly 80% of small HCC nodules do not exhibit elevated AFP levels, and its sensitivity for tumors smaller than 3 cm is less than 25%^31^. The latest AASLD HCC monitoring guidelines have excluded AFP^32^. Therefore, identifying new sensitive molecular markers and therapeutic targets is crucial for improving the early diagnosis and 5-year survival rate of liver cancer.

In recent years, bioinformatics analysis technology as an important research method has provided new avenues for exploring the mechanisms of tumor development and progression by analyzing genomic, transcriptomic, and proteomic data. Our study obtained gene expression data from normal liver tissue and hepatocellular carcinoma tissue from public databases such as GEO and TCGA and identified a gene closely related to hepatocellular carcinoma, AKR1B10, using various bioinformatics analysis methods to determine its biological function and potential regulatory pathways in liver cancer development. Our research first explored the expression pattern of AKR1B10 in HCC, its correlation with clinical pathological features, and its prognostic value through bioinformatics analysis. Our results show that AKR1B10 is significantly upregulated in HCC tissues, with higher expression in male patients in the early stages of the disease, with higher tissue differentiation, severe fibrosis, inflammation in pericancerous liver tissue, or residual foci after surgery. This led us to question whether it affects patient survival status. Subsequently, we conducted a comprehensive prognostic analysis of six tumors with significant overexpression of AKR1B10 in pan-cancer studies through the GSCA database, and the results show that although AKR1B10 exhibits significant expression differences in various tumors, it is only significantly related to poor survival in liver cancer patients, highlighting the importance of AKR1B10 as a prognostic biomarker for HCC. Further COX regression analysis confirmed that it can still be an independent factor predicting HCC prognosis when studied with numerous clinical variables. A systematic review and meta-analysis found that AKR1B10 shows high specificity and sensitivity in HCC diagnosis, especially when used in combination with AFP, the sensitivity and specificity of early HCC diagnosis can reach 0.84 and 0.88^16^. Another large-scale multicenter study followed 273 newly diagnosed HCC patients for two years, finding that serum AKR1B10 levels were significantly related to tumor stage, size, the number of primary tumors, and Child–Pugh classification, and HCC patients with high serum AKR1B10 levels had a significantly lower median survival than those with normal levels^33^. These results support our findings, highlighting the potential importance of AKR1B10 in the diagnosis and treatment of HCC patients.

Although AKR1B10 has significant clinical value in the diagnosis and prognosis assessment of HCC, its specific role and regulatory mechanisms in HCC development are not yet fully clear. To preliminarily clarify the mechanism of action of AKR1B10 in HCC, we further used the GEPIA2 database to extract genes most strongly correlated with AKR1B10 for GSEA enrichment analysis, showing that AKR1B10 mainly interacts with TOLL-like receptors, NOD-like receptors, KEAP1/NFE2L2, and NF-κB transcription factors. Additionally, to explain the potential molecular mechanisms affecting prognosis, we obtained 160 hub genes by intersecting LIHC prognosis-related genes with AKR1B10-related genes and further analyzed the biological processes and signaling pathways of these hub genes using GO functional enrichment and KEGG pathway analysis. The enrichment results suggest that most hub genes are related to biological processes such as regulating redox reactions, glucose-6-phosphate metabolic processes, and astrocyte differentiation. It is well known that these factors are important molecules related to immune and inflammatory regulation. Among them, KEAP1/NFE2L2 is an important pair of regulatory factors, with KEAP1 regulating the stability of NFE2L2 and participating in the regulation of cellular oxidative stress response, while AKR1B10 is an oxidoreductase that participates in metabolic regulation with NADPH as a cofactor. Therefore, we infer that the KEAP1/NFE2L2 pathway may be involved in the normal oxidative stress regulation within hepatocytes. Another transcription factor, NF-κB, has been shown to play different roles at different stages of liver disease: in the early stages of chronic liver disease, the activation of NF-κB may help promote inflammatory responses and cell proliferation, leading to liver tissue damage and fibrosis; as the disease progresses, the overactivation of NF-κB may lead to the malignant transformation of hepatocytes, thereby promoting the development of liver cancer. Our previous study has shown that AKR1B10 is expressed higher in the early stages of HCC, hence we infer that NF-κB can interact with immune response molecules such as TOLL-like receptors, NOD-like receptors, directly or indirectly regulate the transcription of AKR1B10, thus affecting its expression level, jointly controlling the "chronic hepatitis—HCC" evolution. Multiple studies have confirmed that in patients infected with hepatitis C virus (HCV)^34^ and hepatitis B virus (HBV)^35^, high expression of AKR1B10 is an independent prognostic factor predicting HCC development, and AKR1B10 may even be a molecular marker reflecting the progression from steatohepatitis to HCC^36^. Recent studies have revealed that AKR1B10 promotes the proliferation, migration, and invasion of HCC cells through the PI3K/AKT signaling pathway^37^. These findings are consistent with our gene enrichment analysis results, indicating that AKR1B10 may affect the biological characteristics of HCC by regulating inflammation-related signaling pathways, thereby affecting tumor development and patient prognosis.

As is well known, inflammatory responses lead to changes in the number and types of immune cells in the tumor immune microenvironment and the release of cytokines and chemokines, thereby regulating the migration and activation of immune cells. Previous studies have reported that the degree of immune cell infiltration in tumors can affect patient prognosis, and the level of tumor-infiltrating lymphocytes is an independent predictor of patient prognosis^38,39^. As an inflammatory regulator in HCC, AKR1B10's influence on the immune microenvironment of hepatocellular carcinoma was further studied. The results found that AKR1B10 expression in HCC is related to the infiltration patterns of macrophages, dendritic cells and T cells,especially CD8+ T cells. And is associated with immune-promoting factors TNFSF to promote the production of inflammatory factors and the occurrence of inflammatory responses. Macrophages are important anti-tumor immune cells in the immune system, with functions such as phagocytosis and clearance of foreign bodies and regulation of inflammatory responses. Dendritic cells are important antigen-presenting cells in the immune system, capable of capturing and presenting antigens and activating immune responses. T cells protect the body from infection and disease by directly killing pathogens or abnormal cells, regulating the activity of other immune cells, and participating in the formation of immune memory. CD8+ T cells protect the body from infection by recognizing and killing host cells infected with viruses, bacteria, or other parasites. more indirectly indicates that when the high expression of AKR1B10 promotes the occurrence of hepatocellular carcinoma, the number or activity of positive protective factors may increase synchronously in the early stage of the body, thus enhancing the body's ability to clear cells. However, the negative correlation with CD8+ T cells may indicate that in some cases, the increased expression or activity of AKR1B10 may inhibit the function or number of CD8+ T cells, and thus lead to the weakened immune response of the immune system to infection and tumor. This interaction may have an important impact on the immune escape of tumors and immunotherapy. A recent study showed that AKR1B10 led to the increase of immunosuppressive signals in the tumor microenvironment by influencing the polarization of M2-type macrophages, providing favorable conditions for the development of gastric cancer^40^. Wu et al. found that the aldose reductase inhibitor fidarestat can reduce the expression of AKR1B10 in NK cells, thereby promoting NK cell glycolysis and enhancing its killing activity against liver cancer cells^41^. As a co-stimulatory molecule on T lymphocytes, CTLA-4 is involved in the negative regulation of the immune system, which can inhibit the activation and proliferation of T cells, thus weakening the immune response, and plays an important role in the regulation of autoimmunity and anti-tumor immunity. Our study found that the increased expression of AKR1B10 may promote the increase of the expression level of CTLA-4, which may lead to the enhancement of the negative regulation of the immune system and the inhibition of the activation and proliferation of T cells, thereby reducing the immune response ability of the body, and then affecting the development and deterioration of liver cancer. This suggests that by regulating AKR1B10, the metabolic state of immune cells can be affected, thereby affecting their immune killing effect on liver cancer cells, providing insights into the potential immune regulatory role of AKR1B10 in HCC, highlighting its involvement in the regulation of the tumor microenvironment and immune cell responses, but further experimental research is needed to clarify the underlying mechanisms driving these correlations and their potential implications in immune regulation and disease.

ceRNA (competing endogenous RNA) is a complex regulatory network in cells where non-coding RNA and coding RNA interact through miRNA. Understanding this mechanism helps in deepening our research into gene expression and cellular function regulation, opening new doors to understanding gene regulation. This allows us to more comprehensively understand the complex interactions between molecules within cells. In the ceRNA network, mRNA is not only a messenger in protein synthesis but also plays a crucial role as a target gene. Its expression level is strictly regulated by miRNA. miRNA acts as a regulatory factor, controlling the expression levels of target genes' mRNA and lncRNA, thereby affecting cellular functions. lncRNA, by binding with miRNA, regulates miRNA activity, thus affecting miRNA's regulation of other target genes. Based on the LncRNA-miRNA-mRNA regulation model, we used six target gene prediction databases, including LncBase and miRWalk, to infer the specific regulatory network of AKR1B10 in HCC. We found that miR-216a-5p is likely significantly related to AKR1B10 expression. Furthermore, by reverse predicting miR-216a-5p upstream lncRNAs, we identified potential regulatory factors. Among them, DANCR is significantly related to the overall survival rate of liver cancer patients and is highly expressed in liver cancer tissues. In addition, DANCR was expressed at a higher level in advanced HCC. Therefore, we infer that the regulatory network DANCR-miR-216a-5p-AKR1B10 plays a role in hepatocellular carcinoma (HCC). MiR-216a, belonging to the miR-216 family, has been found to play a crucial role in HCV infection. By downregulating the expression of Beclin-1 and Atg5 genes, miR-216a-5p interferes with the host cell's autophagic process, thus impeding HCV replication and spread, which suggests that miR-216a-5p may serve as a potential molecular target for combating HCV infection^42^. Another study at the cellular level confirmed that miR-216a-5p restoration rescued A1BG-AS1 attenuated proliferation, migration and invasion of hepatocellular carcinoma.To be specific,A1BG-AS1 positively regulated the levels of phosphatase and tensin homolog and SMAD family member 7, which were reduced by miR-216a-5p in HCC cells^43^.Recent studies have found that DANCR is significantly upregulated in liver cancer tissues, and high levels of DANCR expression are associated with tumor staging, lymph node metastasis, and prognosis in liver cancer patients^44^. Laboratory studies have found that overexpression of DANCR can promote the proliferation, invasion, and metastasis of liver cancer cells and inhibit apoptosis^45^. In addition, DANCR can regulate various signaling pathways, such as Wnt/β-catenin, PI3K/AKT, etc^46^, participating in the occurrence and development of liver cancer. These findings provide strong evidence for our inferences.

To conclude, The high expression of AKR1B10 in hepatocellular carcinoma (HCC) is not only associated with significant clinical and pathological features but may also influence the progression and prognosis of HCC by activating key signaling pathways and altering the tumor immune microenvironment. Detailed mechanism analysis suggests that it may influence the development and prognosis of HCC by regulating the tumor microenvironment through the underlying DANCR-miR-216a-5p-AKR1B10 axis. These findings provide new insights into the role of AKR1B10 in the pathogenesis of HCC and highlight its value as a potential biomarker and therapeutic target. However, while we have employed extensive cross-validation across multiple databases, it is obvious that reliance solely on bioinformatics analysis presents certain constraints. Thus, the specific mechanisms through which AKR1B10 affects the progression of hepatocellular carcinoma necessitate further elucidation via comprehensive basic experimental research, lending greater depth and validation to these preliminary findings. At the same time, we hope to further verify the correlation between AKR1B10 and the clinical characteristics and prognosis of hepatocellular carcinoma patients through the collection and analysis of peripheral blood, liver tissue and other clinical samples, which will provide more evidence and support for our in-depth understanding of the important role of AKR1B10 in the occurrence and development of hepatocellular carcinoma. Meanwhile, it also provides a new idea and target for the early diagnosis and treatment of hepatocellular carcinoma.

## Conclusions

In summary, high expression of AKR1B10 is closely associated with clinicopathological features in hepatocellular carcinoma, and may promote development and influence prognosis by activating inflammation-related signaling pathways and altering the tumor immune microenvironment through the DANCR-miR-216a-5p-AKR1B10 axis.

## Data Availability

All datasets used and/or analyzed during the current study are available from the public database. The specific website is as follows: GEO database(https://www.ncbi.nlm.nih.gov/geo/); GEO2R(https://www.ncbi.nlm.nih.gov/geo/geo2r) ; TIMER database(https://timer.cistrome.org/); GEPIA database(https://gepia2.cancer-pku.cn); HPA database(https://www.proteinatlas.org/); UCSC database(https://xenabrowser.net/datapages/); GSCA Lite database (https://bioinfo.life.hust.edu.cn/web/GSCA); GSEA (https://software.broadinstitute.org/gsea/msigdb/); LinkedOmics database (https://ww.linkedomics.org) ; miRDB (https://mirdb.org/) ; miRWalk (https://mirwalk.umm.uni-heidelberg.de/) ; Targetscan (https://www.targetscan.org/) ; LncBASE (https://diana.e-ce.uth.gr/lncbasev3) ; miRNet (https://www.mirnet.ca/miRNet/home.xhtml) ; StarBASE (https://rnasysu.com/encori/).
